# Adenosine 2A Receptor Antagonist Prevented and Reversed Liver Fibrosis in a Mouse Model of Ethanol-Exacerbated Liver Fibrosis

**DOI:** 10.1371/journal.pone.0069114

**Published:** 2013-07-18

**Authors:** Dian J. Chiang, Sanjoy Roychowdhury, Katelyn Bush, Megan R. McMullen, Sorana Pisano, Kathryn Niese, Mitchell A. Olman, Michele T. Pritchard, Laura E. Nagy

**Affiliations:** 1 Center for Liver Disease Research, Department of Gastroenterology and Hepatology, Cleveland Clinic, Cleveland, Ohio, United States of America; 2 Department of Pathobiology, Cleveland Clinic, Cleveland, Ohio, United States of America; 3 Department of Molecular Medicine, Cleveland Clinic Lerner College of Medicine, Cleveland, Ohio, United States of America; Institute of Hepatology, Foundation for Liver Research, United Kingdom

## Abstract

The effect of moderate alcohol consumption on liver fibrosis is not well understood, but evidence suggests that adenosine may play a role in mediating the effects of moderate ethanol on tissue injury. Ethanol increases the concentration of adenosine in the liver. Adenosine 2A receptor (A2AR) activation is known to enhance hepatic stellate cell (HSC) activation and A2AR deficient mice are protected from fibrosis in mice. Making use of a novel mouse model of moderate ethanol consumption in which female C57BL/6J mice were allowed continued access to 2% (vol/vol) ethanol (11% calories) or pair-fed control diets for 2 days, 2 weeks or 5 weeks and superimposed with exposure to CCl_4_, we tested the hypothesis that moderate ethanol consumption increases fibrosis in response to carbon tetrachloride (CCl_4_) and that treatment of mice with an A2AR antagonist prevents and/or reverses this ethanol-induced increase in liver fibrosis. Neither the expression or activity of CYP2E1, required for bio-activation of CCl_4_, nor AST and ALT activity in the plasma were affected by ethanol, indicating that moderate ethanol did not increase the direct hepatotoxicity of CCl_4_. However, ethanol feeding enhanced HSC activation and exacerbated liver fibrosis upon exposure to CCl_4._ This was associated with an increased sinusoidal angiogenic response in the liver. Treatment with A2AR antagonist both prevented and reversed the ability of ethanol to exacerbate liver fibrosis.

**Conclusion:**

Moderate ethanol consumption exacerbates hepatic fibrosis upon exposure to CCl_4_. A2AR antagonism may be a potential pharmaceutical intervention to decrease hepatic fibrosis in response to ethanol.

## Introduction

The development of liver fibrosis is a complex and dynamic process involving both the parenchymal and non-parenchymal cells in the liver in response to damage and inflammation [1]. The hallmark of this process is the activation of hepatic stellate cell (HSC), the primary source of extracellular matrix (ECM) production in the injured liver [1]. Alcoholic liver disease (ALD) is one of the major causes of liver fibrosis [2]. Studies in patients with alcoholic cirrhosis find that women are more susceptible to ethanol-induced liver injury and female gender is an independent risk factor for cirrhosis [3]–[5]. Our understanding of ethanol-induced liver fibrosis is largely derived from studies of heavy alcohol consumption in human. However, the role of moderate alcohol consumption in liver fibrosis and its impact on a secondary liver injury is not well understood.

Studies of the effect of moderate alcohol on chronic liver disease and fibrosis are hampered by lack of appropriate animal model. By using 2%(vol/vol) (11% of calories) ethanol feeding in conjunction with CCl_4_ exposure in mice, we established a novel animal model to recapitulate moderate alcohol consumption with a superimposed hepatic toxin. The amount of ethanol intake in this model is equivalent of 2 drinks of alcohol a day in human. We hypothesized that moderate ethanol intake, at a level not sufficient to induce CYP2E1 or cause liver injury by itself, may exacerbate hepatic fibrosis in the setting of a superimposed hepatic injury.

One potential pathway by which moderate alcohol could exacerbate fibrosis is via localized tissue hypoxia and subsequent adenosine receptor activation. Adenosine is a ubiquitously produced signaling molecule with increased concentration in the site of tissue injury and hypoxia [6]. Extracellular adenosine signals through four G–protein coupled adenosine receptors, A1, A2A, A2B, and A3 [6]. Ethanol causes hepatic hypoxia [7] and increases adenosine in the liver by multiple mechanisms including, ethanol metabolism and oxidation, inhibiting the uptake of adenosine via the equilibrative nucleoside transporter, as well as metabolism of AMP via ecto-5′-nucleotidase (CD73) [8]–[10]. The role of adenosine and the downstream pathways of adenosine receptor activation in liver injury and fibrosis are not completely understood. A2AR deficient mice are protected from CCl_4_− or thioacetamide- induced fibrosis [11]. Further, A2AR activation enhances HSC activation [12]. A2AR activation also signals through the PI3K/PKB/Akt in the development of hypoxic preconditioning of hepatocytes [13] and increases angiogenesis in response to injury [14]. On the other hand, A2AR also plays a key role in down-regulating immune response upon injury [15]. Mice deficient in A2AR display elevated and prolonged production of proinflammatory cytokines, including TNFα and IFNγ, in response to challenge with lipopolysaccharide (LPS) [16]. However, the role of adenosine and A2AR activation in the progression of fibrosis is not well studied. Several epidemiological studies have suggested that the consumption of coffee, which contains an adenosine receptor antagonist, caffeine, significantly diminishes hepatic injury and disease progression in patients with chronic liver disease [17].Here we evaluated the preventive, as well as therapeutic effect of A2AR antagonist in a mouse model of ethanol-exacerbated liver fibrosis.

## Materials and Methods

### Materials

CCl_4_ and olive oil were purchased from Sigma-Aldrich (St. Louis, MO). A2AR antagonist (KW-6002) was prepared and provided by Gilead (Foster City, CA). Pair-fed control diet and modified Lieber-DeCarli high-fat diet were purchased from Dyets, Inc (Bethlehem, PA). All primers for quantitative real-time reverse-transcription PCR (qRT-PCR) were synthesized by Integrated DNA Technologies (Coralville, IA). Primary antibodies were purchased from the following companies: Cytochrome P450 2E1 (CYP2E1): Research Diagnostics, Inc (Flanders, NJ); HSC 70:Santa Cruz (Santa Cruz, CA); α-smooth muscle actin (α-SMA, clone 1A6):Sigma-Aldrich (St. Louis, MO), Collagen 1:Southern Biotech (Birmingham, AL), CD31: Gene Tex (Irvine, CA).

### Animals

Female C57BL/6J mice (10–12 week-old) were purchased from Jackson Laboratory. Animals were housed in standard microisolator cages and fed standard laboratory chow (rodent diet #2918, Harlan-Teklad, Madison, WI) prior to initiation of liquid diet feeding. All animal procedures were approved by the Cleveland Clinic Institutional Animal Care and Use Committee.

### Ethanol Feeding and CCl_4_ Administration

We established three independent mouse models to evaluate the response to moderate ethanol consumption and CCl_4_ exposure. In the 72 hour acute CCl_4_ model, mice were fed a control-liquid diet for 2 days, then 1% (vol/vol) ethanol diet for 2 days, then 2% (vol/vol) (11% calories) ethanol diet and one single intraperitoneal (IP) injection of olive oil or CCl_4_ (prediluted1∶3 in olive oil, administered at a dose of 1 µl/g body weight of prediluted CCl_4_ using 100 µl Hamilton syringes and 26G 5/8 inch needles) on day 4. Mice were euthanized 72 hours after injection with CCl_4_. In some experiments, peripheral blood was collected via saphenous vein 24 and 48 hours after injection with CCl_4_. In the 2 week model, the mice were fed with control-liquid diet for 2 days, 1% (vol/vol) ethanol diet for 2 days, then 2% (vol/vol) ethanol diet and IP injection of olive oil or CCl_4_ twice per week for 2 weeks. In the 5 week chronic CCl_4_ model, the mice were fed with control-liquid diet for 2 days, then 1% (vol/vol) ethanol diet for 2 days, then 2% (vol/vol) ethanol diet and an IP injection of olive oil or CCl_4_ twice a week for 5 weeks. For the 2 week and 5 week models, mice were ramped up to the full dose of CCl_4_ over two doses (first injection at 0.25 µl/g body weight, the second at 0.5 µl/g body weight). 72 hours after the last CCl_4_ injection, mice were weighed and anesthetized. Blood was collected from the posterior vena cava by syringe and expelled into EDTA-containing tubes. Livers were excised and mice were then euthanized by exsanguination. Livers were weighed and portions fixed in formalin, frozen in Optimal Cutting Temperature medium (Sakura Finetek USA, Torrance,CA), snap frozen in liquid nitrogen, or stored in RNA*later* (Ambion, Austin, TX) for further analysis. Plasma was separated from whole blood and stored at −80°C.

### A2AR Antagonist Administration

A2AR antagonist (KW-6002) (1 mg/ml in normal saline) or normal saline vehicle as control was given to the mice at a dose of 10 mg per kg of body weight subcutaneously. The dose and route of A2AR antagonist KW-6002 administration was chosen based on previously published data for the pharmacokinetics of this compound [18]. The dosing regimen was chosen to achieve drug concentrations sufficient to antagonize the A2AR, but still maintain specificity for A2AR in mice. Intraperitoneal administration of KW-6002 at 25 mg/kg to C57BL/6J mice reaches C_max_ of 1030 ng/ml at 1.5 hrs; the half-life of KW-6002 is 11.6 hrs (personal communication with Jeff Zablocki at Gilead). KW-6002 is a selective A2AR antagonist in mice with binding affinity of 1.87 nM for A2AR compared to 105.02 nM for A1R [19]. KW-6002 has binding affinity of 151.8 nM for A2AR compared to 11169 nM for A1R, 2701 nM for A2BR and 1939 nM for A3R when assessed in human receptors overexpressed in CHO and HEK293 cells [18]. The molecular weight of KW-6002 is 384.43. Therefore, KW-6002 administration at 10 mg/kg would result in an effective concentration of approximately 1 nM, a concentration highly specific for A2AR. Three treatment protocols were used: 1) To study the effect of A2AR antagonist on the inflammatory and angiogenic cytokine mRNA response, a short time course study was performed with single injection of CCl_4_. Mice were pre-treated with KW-6002 and then challenged with CCl_4_ and euthanized at 4 hour, 8 hour and 24 hour time points. 2) To study the preventive effect of A2AR antagonist, KW-6002 was given one hour prior to each CCl_4_ administration in the 2 week models, and 3) To study the therapeutic effect of A2AR antagonist in resolving established liver fibrosis, KW-6002 was given once daily for 5 days in the last week of the 5 week model.

### Liver Hypoxia: Pimonidazole Adduct Formation

C57BL/6J wild-type mice were allowed free access to diets with increasing concentrations of ethanol to 2% ethanol vol/vol (11% of calories) or pair-fed controls for 4 days. All animals were injected intraperitoneally with pimonidazole (120 mg/Kg) 1 hour before euthanasia. Paraffin-embedded liver sections were de-paraffinized and stained for pimonidazole-adducts using a kit from Hypoxyprobe (Burlington, MA).

### Biochemical Assays

Plasma samples were assayed for ALT and AST using commercially available enzymatic assay kits (Diagnostic Chemicals, Ltd., Oxford, CT) following the manufacturer’s instructions. CYP2E1 activity was determined by measuring the hydroxylation of *p*-nitrophenol in whole liver extract as described [20]. Hepatic collagen content was determined by measuring hydroxyproline concentration in a colorimetric assay. In brief, 300–400 mg tissue was excised from the left and right lobes of the liver, homogenized and hydrolyzed in 6N HCL at 120°C for 16 hours. The hydrolysate was filtered and the amount of hydroxyproline in the liver acid–hydrolysates was determined by colorimetric assay [21], [22].

### qRT-PCR

Total RNA was isolated from liver and 4 µg of total hepatic RNA was reverse transcribed as previously described [23]. Real-time PCR amplification of Col1A1, Col1A2, α-SMA, angiopoietin 1 (ANGP1), TNFα and macrophage inflammatory protein-2 (MIP2) was performed using Brilliant SYBR Green QPCR Master Mix (Stratagene, La Jolla, CA) in an Mx3000p PCR machine (Stratagene). The relative amount of target mRNA was determined using the comparative threshold (*C*_t_) method by normalizing target mRNA *C*_t_ values to those of 18S [24].

### Liver Homogenate and Immunoblotting

Liver homogenates were prepared and protein concentrations were determined for immunoblotting [23]. 35 µg of protein were resolved on 15% polyacrylamide gels and transferred to polyvinylidene fluoride membranes. Membranes were probed with antibodies specific for CYP2E1; HSC 70 was used as loading control.

### Morphological Assessment

For histological analysis of ECM deposition, formalin-fixed tissues were paraffin-embedded, sectioned (5 µm), and stained with Sirius red. The presentation and distribution of α-SMA was studied by using formalin-fixed paraffin embedded liver sections (5 µm) via immunochemical staining [24]. Frozen liver sections were used for immunostaining of CD31, a marker for vascular endothelial cells and collagen 1 [25], [26]. Slides were coded before examination and viewed by a single investigator who was blinded to the treatment. All images presented in the results are representative of at least 3 images per liver and 4 to 6 mice per experimental condition. Semi-quantification of positive staining was performed using ImagePro plus software (Media Cybernatics, Silver Spring, MD).

### Statistical Analysis

Values reported are means +/− SEM. The data were analyzed by general linear models procedure (SAS, Carey, NC) followed by least square means or Tukey’s analysis of differences between groups. *P* values of less than 0.05 were considered significant.

## Results

### Moderate Ethanol Intake did not Induce CYP2E1 or Affect Hepatotoxicity of CCl_4_

**Figure 1:**
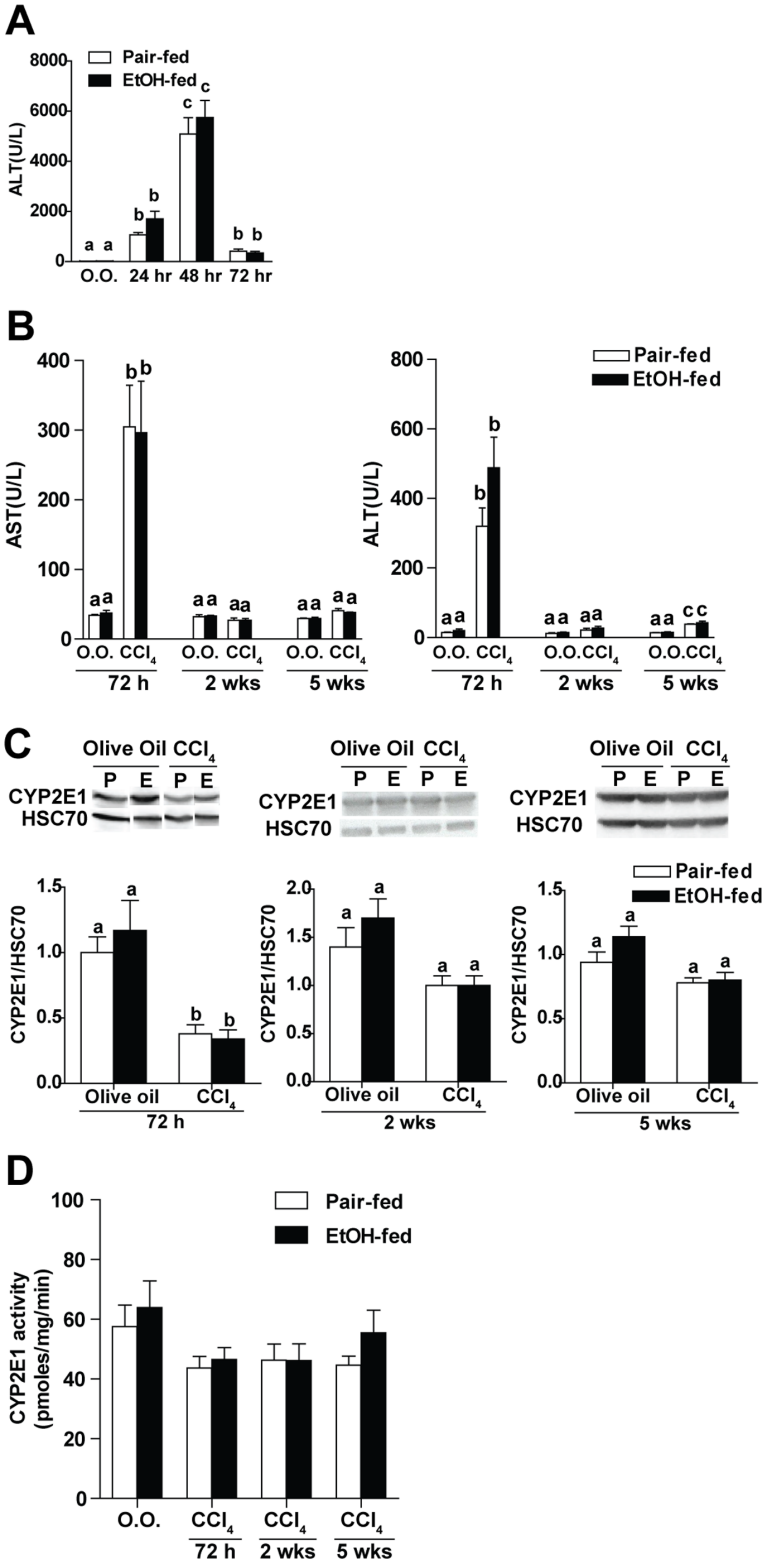
Effect of moderate ethanol intake on CYP2E1 and CCl_4_ hepatotoxicity. C57BL/6J mice were allowed free access to 2% (vol/vol) (11% calories) ethanol diet or pair-fed control diet and then exposed to intraperitoneal CCl_4_ injections over 72 hours, 2 weeks or 5 weeks. (**A**) Plasma ALT was measured in the acute time course to assess hepatic injury. (**B**) Plasma AST and ALT were measured at different time points. (**C**) CYP2E1 protein level in liver was measured by Western blot using whole liver extracts. HSC 70 was used as loading control. Insets show representative image of CYP2E1 Western blots. (**D**) Activity of CYP2E1 in liver was measured by the hydroxylation of *p*-nitrophenol in whole liver extracts. Values represent means ± SEM. n = 4 in pair-fed diet, n = 6 in ethanol-fed diet. Values with different alphabetical superscripts were significantly different from each other, p<0.05.

The rate-limiting step for CCl_4_ toxicity is its bioactivation to CCl_3_ through CYP2E1 [20]. Since CYP2E1 is induced by high concentrations of ethanol [27], any effect of ethanol may be confounded by the increased bioactivation of CCl_4_. Here we made use of a novel mouse model using 2%(vol/vol) ethanol diet and exposed the mice to CCl_4._ Ethanol feeding alone did not increase AST or ALT, compared to pair-fed mice, at any time point studied. Plasma AST and ALT activity increased after injection of CCl_4_. ALT in the plasma reached a maximum at 48 hr after CCl_4_ exposure (Figure 1A). However, there was no difference in plasma ALT and AST activity between mice on ethanol or pair-fed diet after exposure to CCl_4_ (Figure 1A and 1B). Further, the 2%(vol/vol) ethanol diet was not sufficient to increase CYP2E1 protein level compared to pair-fed controls at 72 hr, 2 weeks or 5 weeks (Figure 1C). In addition, there was no difference in the CYP2E1 enzyme activity in liver in response to ethanol or CCl_4_ challenge (Figure 1D). Taken together, these data demonstrated that 2%(vol/vol) ethanol intake did not induce CYP2E1, cause direct liver toxicity or increase the hepatic toxicity of CCl_4._

### Moderate Ethanol Intake Enhanced Activation of HSC in Response to CCl_4_

**Figure 2:**
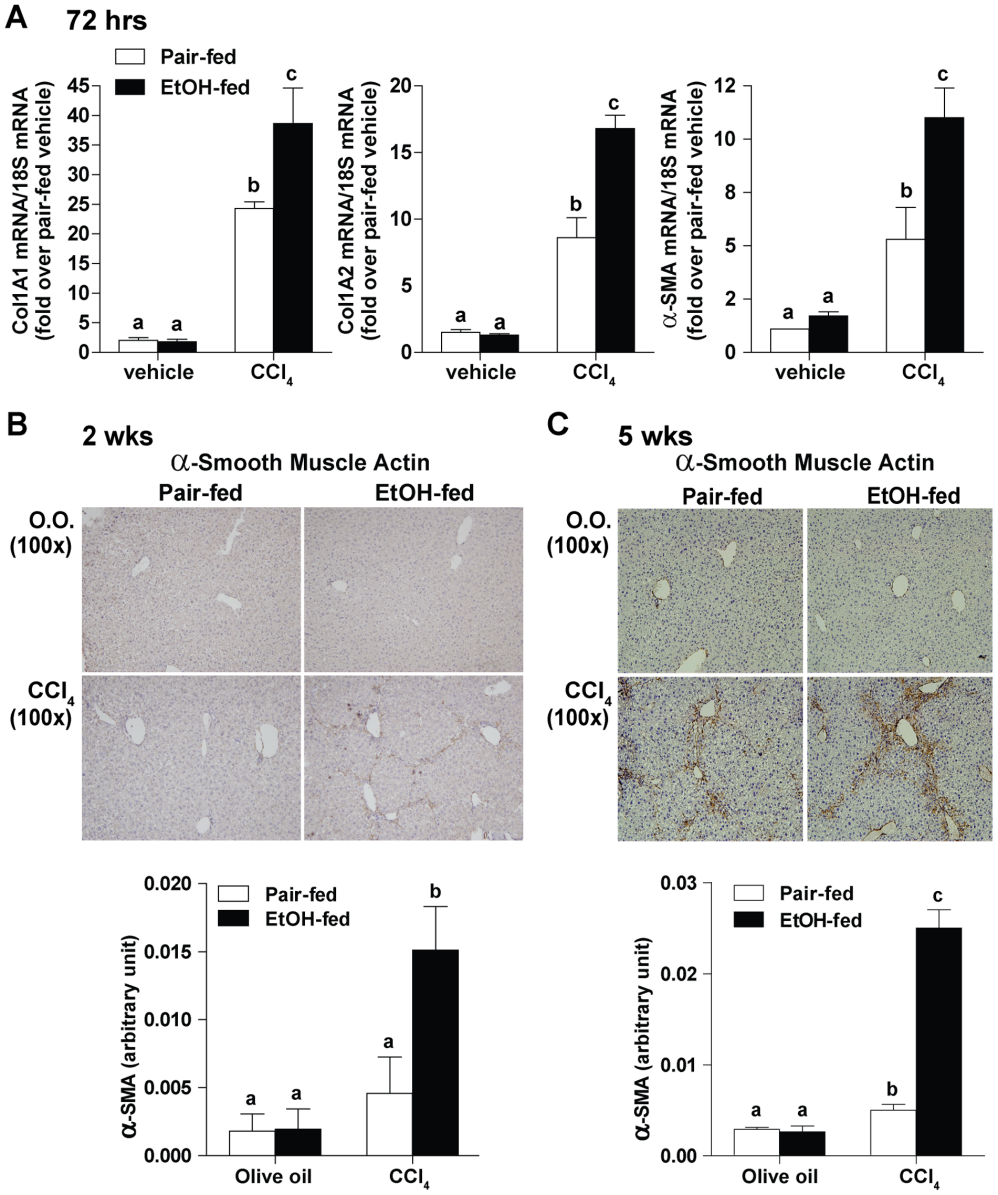
Effect of moderate ethanol intake on HSC activation in response to CCl_4_. C57BL/6J mice were allowed free access to 2% (vol/vol) ethanol diet or pair-fed control diet and then exposed to intraperitoneal CCl_4_ injections over 72 hours, 2 weeks or 5 weeks. (**A**) Hepatic accumulation of Col1A1, Col1A2 and α-SMA mRNA was used as intermediate biomarkers of HSC activation and fibrosis at 72 hours. (**B, C**) Paraffin-embedded liver sections were de-paraffinized followed by α-SMA staining at 2 weeks (**B**) and 5 weeks (**C**). Representative images are shown. Morphometric analysis with Image-Pro-Plus software was used for semi-quantification. Values represent means ± SEM. n = 4 in pair-fed diet, n = 6 in ethanol-fed diet. Values with different alphabetical superscripts were significantly different from each other, p<0.05.

Increased HSC activation in the liver promotes the accumulation of ECM and is essential in the initiation and perpetuation of liver fibrosis. Activated HSC increase expression of mRNA of Col1A1, Col1A2 and α-SMA. These mRNA are considered intermediate biomarkers for eventual development of fibrosis. Ethanol feeding alone had no effect on the expression of these intermediate biomarkers of HSC activation and fibrosis (Figure 2A). Challenge with CCl_4_ increased expression of Col1A1, Col1A2 and α-SMA mRNA in both pair-fed and ethanol-fed mice compared to olive oil controls (Figure 2A); however, this increase was greater in ethanol-fed mice compared to pair-fed mice (Figure 2A). Consistent with the mRNA data, ethanol (2% (vol/vol)) intake alone did not increase α-SMA positive HSC in the liver at 2 weeks (Figure 2B) or 5 weeks (Figure 2C). As expected, CCl_4_ exposure increased α-SMA positive HSC in the liver of both pair- and ethanol-fed mice; ethanol-feeding resulted in a four-fold greater increase of α-SMA staining in the liver sinusoid compared to pair-fed mice at both 2 weeks (Figure 2B) and 5 weeks (Figure 2C) of CCl_4_ administration.

### Moderate Ethanol Intake Exacerbated Liver Fibrosis

**Figure 3:**
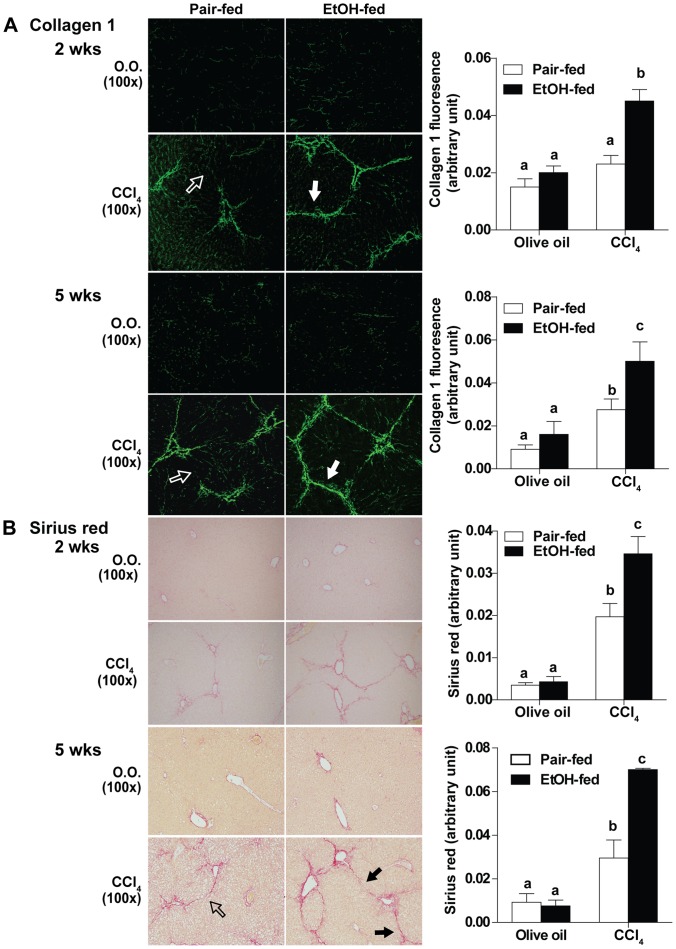
Progression of fibrosis with moderate ethanol intake and superimposed CCl_4_ liver injury. C57BL/6J mice were allowed free access to 2% (vol/vol) ethanol diet or pair-fed control diet and then exposed to intraperitoneal CCl_4_ injections for 2 weeks or 5 weeks. (**A**) Collagen 1 staining was performed on frozen liver sections at 2 weeks and 5 weeks. (**B**) Paraffin-embedded liver sections were de-paraffinized followed by Sirius red staining to assess ECM deposition at 2 weeks and 5 weeks. Representative images are shown. (Solid arrow: immunostaining pattern consistent with bridging fibrosis. Open arrow: immunostaining pattern less than bridging fibrosis.) Morphometric analysis with Image-Pro-Plus software was used for semi-quantification. Value represents mean ± SEM. n = 4 in pair-fed diet, n = 6 in ethanol-fed diet. Values with different alphabetical superscripts were significantly different from each other, p<0.05.

The accumulation of ECM and resultant change in the architecture of hepatic sinusoid is the hallmark of liver fibrosis. The ethanol (2% (vol/vol)) diet alone did not increase collagen 1 positive staining in the liver compared to mice pair-fed control diet (Figure 3A). CCl_4_ increased collagen 1 expression in the liver sinusoid at both 2 weeks and 5 weeks. After both 2 and 5 weeks of CCl_4_ exposure, collagen 1 positive staining was increased in ethanol-fed mice compared to pair-fed mice (Figure 3A). Similarly, CCl_4_ increased Sirius red staining compared to olive oil controls in both pair- and ethanol-fed mice. While ethanol alone did not increase Sirius red positive staining compared to pair-fed diet (Figure 3B), after CCl_4_ exposure, Sirius red staining was enhanced by ethanol feeding compared to pair-fed controls (Figure 3B). Compared to the 2-week time point, ECM deposition in the sinusoidal space is even more prominent by 5 weeks, with progression to bridging fibrosis (Figure 3B).

### A2AR Antagonist Decreased Activated HSC and Liver Fibrosis

**Figure 4:**
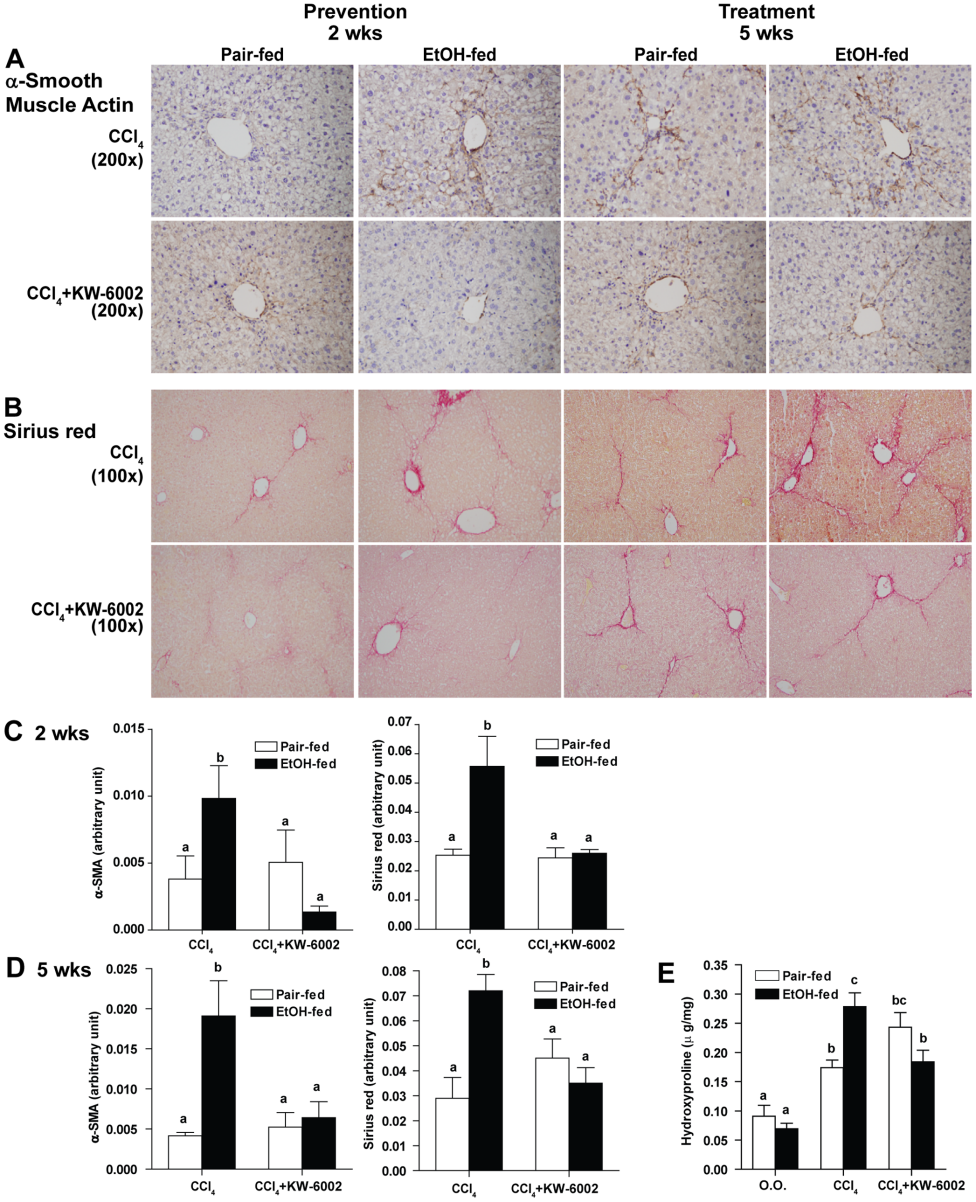
Effect of A2AR antagonist in prevention and treatment of CCl_4_-induced HSC activation and liver fibrosis. C57BL/6J mice were allowed free access to 2% (vol/vol) ethanol diet or pair-fed control diet and exposed to intraperitoneal CCl_4_ injections with KW-6002 for 2 weeks in the prevention model or with KW-6002 in the last week of the 5 weeks treatment model. (**A**) α-SMA was used as a marker for activated HSC in the 2 week and 5 week models. Representative images are shown. (**B**) ECM deposition was measured by Sirius red staining in the 2 week and 5 week models. Representative images are shown. (**C, D**) Morphometric analysis with Image-Pro-Plus software was used for semi-quantification. Values represent means ± SEM. n = 4 in pair-fed diet, n = 6 in ethanol-fed diet. Values with different alphabetical superscripts were significantly different from each other, p<0.05. (**E**) Hepatic hydroxyproline concentration was measured using a colorimetric assay in liver acid–hydrolysates in the 5 week model. Values represent means ± SEM. n = 3–8 in each group. Values with different alphabetical superscripts were significantly different from each other, p<0.05.

To test the hypothesis that A2AR activation contributes to the worsening of liver fibrosis from moderate ethanol consumption, mice were treated with A2AR antagonist KW-6002 in the chronic models. Treatment of A2AR antagonist did not alter the degree of liver injury as measured by plasma AST and ALT levels at 2 week and 5 week time points (Table 1). Treatment with A2AR antagonist had no effect in CCl_4_-mediated increase in α-SMA in pair-fed mice, but prevented the ethanol-induced increase in α-SMA staining in ethanol-fed mice after exposure to CCl_4_ (Figure 4A, C). KW-6002 also prevented the ethanol-exacerbated ECM deposition, assessed by Sirius red stain at week 2 (Figure 4B, C). To determine if A2AR antagonist may reverse fibrosis, KW-6002 was given daily at the last week of our 5-week model as a therapeutic agent. KW-6002 decreased α-SMA staining in ethanol-fed mice after exposure to CCl_4_ (Figure 4A, D) and Sirius red positive ECM deposition in ethanol-fed mice with CCl_4_ exposure, indicating that it was able to reverse fibrosis after it was established (Figure 4B, D). Hydroxyproline concentration in the liver was also measured to quantify hepatic fibrosis. Moderate ethanol feeding alone did not increase hydroxyproline concentration in the liver compared to pair-fed mice (Figure 4E). CCl_4_-exposure increased hydroxyproline concentration in the liver of both ethanol- and pair-fed mice; however, hydroxyproline was higher in mice exposed to ethanol and CCl_4_ compared to pair-fed mice treated with CCl_4_. Treatment with KW-6002 decreased hydroxyproline concentration in ethanol-fed mice after exposure to CCl_4,_ but had no effect in pair-fed mice exposed to CCl_4_ (Figure 4E). These data demonstrated that A2AR antagonist decreased HSC activation, prevented and reversed liver fibrosis.

**Table 1:** Effect of A2AR antagonist on liver injury.

	Pair-fed+ CCl_4_				EtOH-fed+ CCl_4_			
	Saline		KW-6002		Saline		KW-6002	
	AST	ALT	AST	ALT	AST	ALT	AST	ALT
2 wk	27.3±3.1	21.9±4.1	31.7±4.1	27.6±6.1	27.2±2.2	27.1±5.0	39.9±4.3	37.6±5.1
5 wk	40.6±3.0	38.4±1.4	42.4±3.7	34.4±3.3	49.1±10.9	42.1±4.9	50±8.3	51±4.7

Plasma AST and ALT levels in pair-fed and ethanol-fed C57BL/6J mice exposed to CCl_4_ with KW-6002 or saline control at the 2 week and 5 week time points. Values represent mean ± SEM. n = 4 in pair-fed diet, n = 6 in ethanol-fed diet.

### Ethanol Induced Local Hypoxia and Enhanced Sinusoidal Angiogenesis in Response to CCl_4_

**Figure 5:**
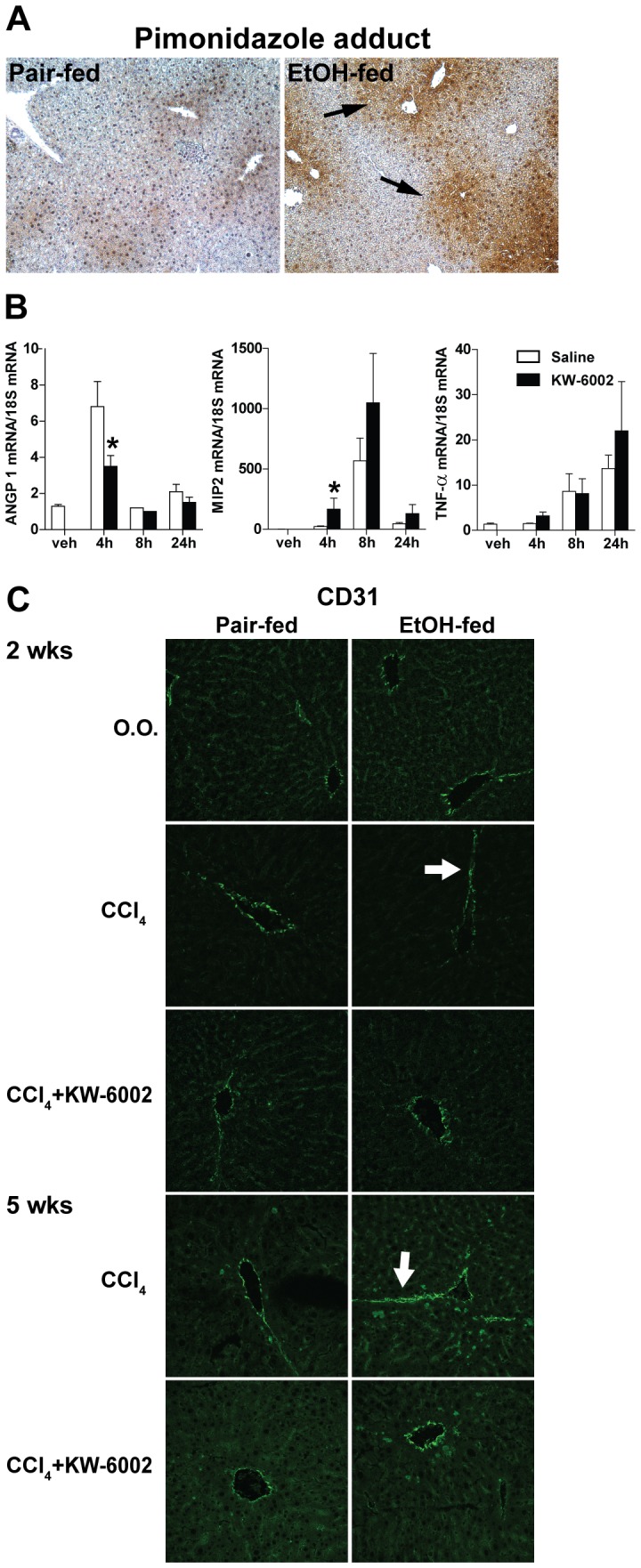
Effect of A2AR antagonist on hepatic angiogenic response and sinusoidal capillarization. (**A**) C57BL/6J wild-type (WT) mice were allowed free access to diets with increasing concentrations of 2% (vol/vol) ethanol or pair-fed controls for 4 days. All animals were injected intraperitoneally with pimonidazole (120 mg/Kg, PMD) 1 hour before euthanasia. Paraffin-embedded liver sections were de-paraffinized and stained for PMD-adducts. Representative images are shown. n = 4 in pair-fed diet, n = 6 in ethanol-fed diet. (Solid black arrow: increased pimonidazole adducts accumulation.) (**B**) A short time course study using single injection of CCl_4_ with pretreatment of KW-6002 or saline in C57BL/6J wild-type (WT) mice. ANGP1, TNF-α and MIP2 mRNA in liver was measured after KW-6002 administration as an intermediate marker of angiogenesis and inflammation. (* indicates statistical significance between KW-6002 and saline control, p<0.05) (**C**) C57BL/6J mice were allowed free access to 2% (vol/vol) ethanol diet or pair-fed control diet and then exposed to intraperitoneal CCl_4_ injections with KW-6002 for 2 weeks in the prevention model or with KW-6002 in the last week of the 5 weeks treatment model. Frozen liver sections were used for CD31 immunofluorescent staining to study sinusoidal capillarization and angiogenic response. (Solid arrow: increased CD31 expression in sinusoidal space.) Representative images are shown. n = 4 in pair-fed diet, n = 6 in ethanol-fed diet.

Chronic heavy ethanol feeding results in localized hypoxia in the liver [7]. If moderate ethanol also induces hepatic hypoxia, then pimonidazole adduct formation, a measure of tissue hypoxia, should be elevated. Indeed, increased tissue hypoxia was seen in mice exposed to moderate ethanol feeding as measured by the accumulation of pimonidazole adducts (Figure 5A).The expression of ANGP1 mRNA, an intermediate marker of hepatic angiogenesis, was measured at different time points after single CCl_4_ injection in mice pre-treated with saline or KW-6002. CCl_4_ increased ANGP1 mRNA level at 4 hours. Treatment of mice with KW-6002 prior to exposure to CCl_4_ prevented this increase in ANGP1 mRNA level in liver (Figure 5B). On the other hand, treatment of mice with KW-6002 prior to challenge with CCl_4_ increased inflammatory cytokine responses, consistent with the anti-inflammatory effects of A2AR activation (Figure 5B). CD31, a cell surface marker for vascular endothelial cells, was used as an indicator of an angiogenic response. Ethanol feeding alone did not increase the CD31 positive staining compared to pair-fed diet. However, enhanced CD31 staining in the liver sinusoid was observed in the mice exposed to 2%(vol/vol) ethanol feeding and CCl_4_ compared to pair-fed mice exposed to CCl_4_ (Figure 5C). Interestingly, CD31 positive staining in the sinusoid was diminished with KW-6002 administration (Figure 5C). These data demonstrate that ethanol increased sinusoidal angiogenic response upon exposure to CCl_4_ and A2AR antagonism prevented the ethanol-induced sinusoidal angiogenic response in liver.

## Discussion

Liver fibrosis is a wound healing response to chronic liver injury. The initiation and maintenance of fibrosis is a dynamic process that is characterized by the accumulation of collagens and ECM [1]. Alcohol intake causes hepatocyte injury, which promotes the subsequent activation of HSC, the major cellular source of collagens and ECM in the liver. Acetaldehyde, a reactive metabolite of ethanol directly regulates gene expression in HSC and up-regulate collagen production in the liver [28]. In addition, alcohol disrupts the gut barrier function, elevating LPS concentrations in the liver [29]. LPS can further enhance HSC activation through up-regulation of TGF-β signaling [28]. Activation of Kupffer cells, the resident macrophage in the liver, also leads to production of profibrotic cytokines and chemokines [30]. In addition to the direct liver toxicity, alcohol may also contribute to the progression of fibrosis through disruption of the wound healing response and activation of HSC. Despite the likely contributions of alcohol to hepatic fibrosis, the effect of subclinical/moderate alcohol consumption in the setting of superimposed liver injury is not well understood. There are conflicting findings regarding the impact of moderate alcohol consumption on the progression of chronic liver diseases, particularly from observational studies in patients with non-alcoholic chronic liver disease [31]–[33]. Results between these different studies are difficult to compare due to variation in the definition and quantification of alcohol intake, as well as other dietary confounders. Therefore, a well-characterized animal model would be instrumental in clarifying the role of moderate alcohol consumption in chronic liver diseases.

Given the variable clinical presentation and dynamic nature of alcoholic liver disease, it has been difficult to reproduce the complex process in experimental models [34]. Carbon tetrachloride (CCl_4_)-induced injury is one of the most well studied animal models for hepatic fibrosis. While some previous studies have used ethanol feeding along with CCl_4_ exposure to study the synergistic effect of ethanol [34], the impact from ethanol is confounded by the induction of CYP2E1 in liver and increased liver toxicity from enhanced CCl_4_ metabolism. To address this issue, Gao and colleagues developed a model which titrated the dose of CCl_4_ to provide equivalent initial hepatic toxicity, assessed by AST/ALT, in the ethanol-fed and control mice [35]. In our study, we characterized a novel mouse model in which a low concentration of ethanol was selected for feeding, in order to generate a subclinical model of alcohol exposure that did not result in the induction of CYP2E1 or hepatocyte injury. This model therefore eliminates any potential confounding effects of due to differential bioactivation of CCl_4_in ethanol-fed compared to control mice. The comparable bioactivation of CCl_4_ in ethanol- and pair-fed mice was reflected in the equivalent initial hepatoxicity observed in response to challenge with CCl_4_, reflected in the equal rise in ALT and AST in the plasma. Therefore, this model recapitulates the subclinical situation of moderate alcohol consumption. Moderate ethanol intake alone did not increase ECM deposition or induce liver fibrosis; however, upon challenge with a hepatotoxin, moderate ethanol feeding exacerbated liver fibrosis_._ These data demonstrate a synergistic effect of ethanol and CCl_4_ in liver fibrosis and suggested that moderate alcohol intake may exacerbate liver fibrosis in the setting of a superimposed liver injury or stress.

HSC play a key role in the initiation and progression of liver fibrosis and are the major source of ECM [1]. The exacerbation of liver fibrosis observed here was associated with enhanced HSC activation. One possible mechanism for this increased sensitivity of HSC to activation after moderate alcohol feeding is a “priming” of the HSC. Priming of the HSC is likely to occur in response to localized hypoxia in the liver during ethanol metabolism, with a resultant generation of adenosine [12], as well as additional angiogenic and chemotactic mediators [36]. In our study, moderate ethanol intake caused local hypoxia and increased the angiogenic response in the liver. Our data indicated that moderate ethanol exposure and the “priming” from ethanol-induced hypoxia may exacerbate fibrosis response to subsequent CCl_4_ exposure. Enhanced angionenic response and altered angioarchitecture in the liver correlate with the progression of fibrosis in chronic liver diseases [37]. Hypoxia-induced angiogenic cytokines VEGF and ANGP-1 also up-regulate the activation and migration of HSC and thus contribute to the initiation and maintenance of fibrosis [38], [39].The consequences of the enhanced angiogenic responses are increased vascular permeability and local circulation, which leads to increased oxygen delivery, recruitment of inflammatory cells and bone marrow derived endothelial cells. The subsequent change in angioarchitecture sustains the progression of fibrosis. It has been reported that the establishment of pathologic angioarchitecture is inversely associated with the reversal of liver fibrosis in humans [37]. Our data indicated that moderate ethanol may enhance the hepatic angiogenic reponse upon superimposed liver injury and thus exacerbate progression of fibrosis.

Adenosine is an endogenous regulator of tissue repair in the setting of cell injury and tissue hypoxia. It is produced from the dephosphorylation of adenosine tri-, di-, and monophosphates [40], as well as from the degradation of nucleic acids during cellular injury and apoptosis [41]. The metabolism of ethanol can increase adenosine production via increased ATP hydrolysis during the formation of Acetyl-CoA [42], ethanol metabolism and oxidation [8], inhibiting the uptake of adenosine via the equilibrative nucleoside transporter [9], as well as metabolism of AMP via ecto-5′-nucleotidase (CD73) activity [10]. Adenosine concentrations in the liver are increased in animal models of alcoholic liver injury and both ethanol and CCl_4_ exposure increase adenosine release in an *ex vivo* model of liver slices [11]. While it is technically possible to measure adenosine in the circulation, the physiological relevance of these measures is questionable, since the half-life of adenosine in the circulation is on the order of seconds, due to the presence of adenosine metabolizing enzymes, such as adenosine deaminase, in the serum. Therefore, we utilized a specific A2AR antagonist to delineate the role of adenosine in moderate ethanol intake in this study. We demonstrated that A2AR antagonist administration attenuated the HSC activation in the setting of moderate ethanol intake and CCl_4_ exposure *in vivo*. Treatment with A2AR antagonist not only prevented the progression of fibrosis in the 2-week model but also reversed the fibrosis after it was established at 5 weeks of CCl_4_ exposure. The anti-fibrotic effect of the A2AR antagonist was associated with attenuation of HSC activation and attenuated hepatic angiogenic response. Interestingly, in contrast to previous studies in A2AR^−/−^ mice [11], pharmacological A2AR antagonism after fibrosis was established was not sufficient to completely reverse CCl_4_-induced fibrosis in control mice, but was sufficient to ameliorate the exacerbation of fibrosis by ethanol. These data suggest that the primary mechanism by which moderate ethanol exacerbates fibrosis is via A2AR-mediated mechanisms.

The primary contribution of the A2AR to the exacerbation of fibrosis by ethanol may be due to increases in adenosine itself and/or increases in the expression of adenosine receptors. In addition to an increase in adenosine, ethanol and/or CCl_4_ exposure may also increase expression of A2A receptors in the liver. While A2A receptor expression is increased by LPS, TNFα and other agents that stimulate NFκB (42–45), the expression of mRNA for A1, A2A or A2B receptors was not affected by ethanol or CCl_4_ exposure (data not shown). Taken together, these data suggest that the contribution of A2A receptors during moderate ethanol feeding was not a result of increased A2AR expression in the liver but rather an increase in A2AR activation through increased adenosine.

Adenosine influences responses in a number of wound healing models, including liver [10], kidney [43], lung [44] and skin [45]. Adenosine inhibits PDGF-induced HSC chemotaxis in a dose-dependent manner. This inhibition is mediated via the A2AR [12]. Adenosine also up-regulates the production of TGFβ and collagen I mRNA in HSC *in vitro* [12]. Further, activation of A2AR in HSCs increases collagen 1 expression via cAMP/ERK1/2- dependent pathway and collagen 3 expression via p38 MAPK-dependent pathway [46]. In addition, adenosine may stimulate macrophages to produce VEGF [38], [47] and promote recruitment of bone-marrow derived endothelial precursor cells [25], thus contributing to angiogenesis and wound healing response in tissue injury. Further, A2AR deficient mice are protected from CCl_4_-induced liver fibrosis and A2AR antagonism, when initiated prior to and maintained during CCl_4_ exposure, also reduces hepatic fibrosis [11]. In addition to the anti-fibrotic effect in liver injury, A2A receptor blockade or deletion also diminishes fibrocyte accumulation in the skin in a murine model of scleroderma [45]. On the other hand, administration of A2A receptor agonist prevents kidney fibrosis in a rat model of glomerulonephritis [43] and improves lung function in a model of acute lung injury in rats [44]. The anti-fibrosis effect in the kidney is attributed to the marked decrease in macrophage infiltration and macrophage-linked glomerular damage mediators [43]. In the setting of acute lung injury, the improvement in lung function is a result of increased alveolar fluid clearance via up-regulation of α-epithelial sodium channel in lung epithelial cells and decreased inflammatory cell infiltration [44]. Therefore, the benefits from A2A receptor activation in these two studies are associated, at least in part, with a decrease in inflammation and injury. Importantly, the difference between the model of inflammation-induced injury and fibrosis in the kidney and CCl_4_-induced liver injury is the role of the inflammatory response. In the CCl_4_ model, the primary driver of liver fibrosis is hepatocyte injury and necrosis. Therefore, the contribution of the anti-inflammatory effects of A2AR would likely be less important in CCl_4_-mediated fibrosis, compared to glomerulonephritis in kidney.

Our results suggested that A2AR antagonist not only prevents the progression of fibrosis, but also serves as a therapeutic agent for ethanol-exacerbated liver fibrosis. While the exact mechanism is not clear, the beneficial effect of coffee consumption in observational studies of chronic liver diseases [17] may therefore be related to the presence of caffeine in coffee, which acts as an A2AR antagonist. Sustained activation of HSC is required for the maintenance and progression of fibrosis. The anti-fibrotic effect from A2AR antagonist is, at least in part, related to the decreased activation of HSC. In addition, A2AR antagonist may decrease the fibrosis through normalization of angiogenic response. Another mechanism by which A2AR antagonist may attenuate liver fibrosis is through enhancing the anti-fibrotic effect of NK cells [15].

In summary, this study characterized a novel mouse model to study the effect of moderate ethanol intake in liver fibrosis and demonstrated the synergistic effect of moderate ethanol and CCl_4_. This animal model will be useful to further delineate the interaction of moderate alcohol consumption and subclinical liver injury. The ethanol-exacerbated fibrosis was, at least in part, due to a contribution of hypoxia and A2AR activation, evidenced by the ability of an A2AR antagonist to decrease ethanol-induced HSC activation, fibrosis and dysregulated angiogenesis. The study provides a rationale for A2AR antagonist-based therapy in ethanol-induced liver fibrosis.
